# Durable response to low-dose pralsetinib in a renal insufficient patient with NSCLC harboring concurrent *CCDC6-RET, LINCO1264-RET*, and *SEMA5A-RET* fusions: A case report

**DOI:** 10.1097/MD.0000000000031480

**Published:** 2022-11-25

**Authors:** Linping Gu, Wenxiang Ji, Yunhua Xu, Yuchen Han, Hong Jian

**Affiliations:** a Department of Shanghai Lung Cancer Center, Shanghai Chest Hospital, Shanghai Jiao Tong University, Shanghai, China; b Department of Pathology, Shanghai Chest Hospital, Shanghai Jiao Tong University, Shanghai, China.

**Keywords:** NSCLC, pralsetinib, renal failure, RET fusion, RET tyrosine kinase inhibitors

## Abstract

*RET*-rearranged fusions have been considered as oncogenic drivers in 1% to 2% of non-small cell lung cancers (NSCLC). ARROW study has demonstrated a new selective RET tyrosine kinase inhibitors (TKIs) shows remarkable and durable responses in *RET*-rearranged NCSLC. In this study mainly recruited patients with common fusion partners *KIF5B* and *CCDC6*. There is still a lack of definitive conclusions about effective of rare RET fusion variants to anti-RET therapies.

**Case report:**

A Chinese 58-year-old female renal insufficient patient with no history of smoking was diagnosed as stage IIIA (T2N2M0) lung adenocarcinoma. Next-generation sequencing targeting 520 cancer-related genes was performed on the pleural effusion samples and revealed 2 novel RET fusions LINCO1264-RET and SEMA5A-RET, concomitant with a common CCDC6-RET.

**Management and outcome:**

The patient was first treated with multiple lines of chemotherapy and switched to lenvatinib but failed to respond. Due to renal insufficiency, she subsequently received pralsetinib with gradually reduced dosages (400 mg-300 mg-200 mg-100 mg qd) and achieved a partial response (PR) lasting for more than 10 months, accompanied by the declined allele frequencies of all 3 RET fusions.

**Discussion/conclusions:**

We reported the first case of the pralsetinib efficacy in NSCLC with 3 concurrent RET fusions. Our case also indicates the sensitivity of the newly identified RET fusions to this RET selective inhibitor pralsetinib, and highlights the low-dose treatment option for patients with renal insufficient background.

## 1. Introduction

Mapping to chromosome 10q11.2, *RET* gene encodes a receptor tyrosine kinase comprised of an extracellular domain, a transmembrane region and an intracellular kinase domain. Genomic rearrangements of *RET* gene can form chimeric tyrosine kinase fusion proteins that often confer the constitutive oncogenic RET activation if the intact kinase domain is retained.^[1]^ *RET* fusions occur in 1% to 2% of non-small cell lung cancers (NSCLC) and have been established as oncogenic drivers in this disease.^[2]^

To date, a number of clinical studies have investigated a variety of multikinase inhibitors with anti-RET activity, such as vandetanib, cabozantinib and alectinib, in patients with *RET*-rearranged lung cancer. However, the objective response rate (ORR) (16%–47%) and median progressive-free survival (mPFS) (2.3–7.3 months) are inferior to that seen in other oncogene-addicted NSCLC with targeted therapies, such as EGFR, ALK and ROS1.^[3]^ Moreover, it has been reported that the responsiveness of different *RET* fusion partners varies to the multi-target inhibitors.^[4]^ More recently, a new generation of highly selective RET tyrosine kinase inhibitors (TKIs), namely selpercatinib and pralsetinib, has been developed and demonstrated remarkable and durable responses in *RET*-rearranged NCSLCs, driving the recent FDA approval of the drugs.^[5]^ However, in these clinical trials, recruited patients mainly harbored the common *RET* fusion partners *KIF5B* and *CCDC6*. It remains elusive how other rare partners respond to these selective RET TKIs. Thus, there is still a lack of definitive conclusions on the potentially different responsiveness of diverse *RET* fusion variants to anti-RET therapies. Herein, we described a recurrent NSCLC patient who harbored 2 novel *RET* fusions *LINCO1264-RET* and *SEMA5A-RET* concomitant with a common *CCDC6-RET*. The refractory heavily-pretreated patient had chronic renal insufficiency and received pralsetinib with gradually reduced dosages. She achieved a partial response (PR) lasting for more than 9 months. We present the following case in accordance with the CARE reporting checklist.

## 2. Case presentation

**Figure 1.**
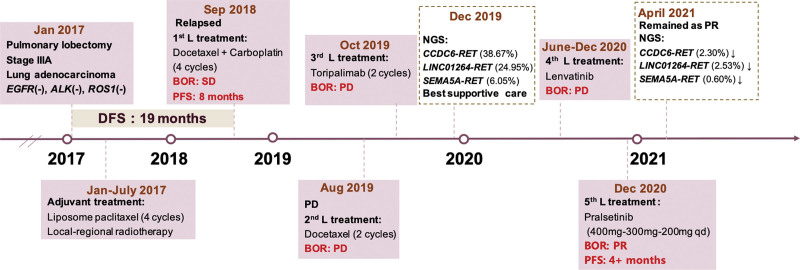
Timeline of the patient’s treatment history. BOR = best overall response, DFS = disease-free survival, NGS = next-generation sequencing, PD = progressive disease, PFS = progressive-free survival, PR = partial response.

A Chinese 58-year-old female never-smoker was referred to our clinic due to the tumor in the right lower lung field identified by a CT scan. Any other sign of metastasis did not discover, Eastern Cooperative Oncology Group (ECOG) performance status 1. The patient had no family history of cancer but had renal insufficiency due to the hydronephrosis (creatinine: 130 umol/L; creatinine clearance: 30 mL/min). In Jan 2017, she had a thoracoscopic lobectomy of the right lower lobe with systemic lymph node dissection. Postoperative histopathological and genetic tests indicated a stage IIIA (T2N2M0) lung adenocarcinoma with negative *EGFR, ALK*, and *ROS1*. Due to the history of renal insufficiency, the patient subsequently received adjuvant treatment with liposome paclitaxel for 6 cycles and local-regional radiotherapy in July 2017 (Fig. 1). After a disease-free survival of 19 months, the patient presented with right pleura thickening accompanied by increased FDG metabolism. She was treated with docetaxel plus carboplatin for 4 cycles and achieved a stable disease lasting for 8 months. In August 2018, the patient had shortness of breath, and was found with massive right pleural effusion and multiple nodules in the right pleura. She received monotherapy of docetaxel rechallenge for 2 cycles but the disease progressed. Then she was switched to a progressive disease-1 inhibitor toripalimab for 2 cycles but developed progressive disease again with the increased creatinine (up to 182 umol/L) (Fig. 1). Toripalimab was held on admission. The renal function of the patient improved significantly and achieved a baseline of serum creatinine (130 umol/L).

**Figure 2.**
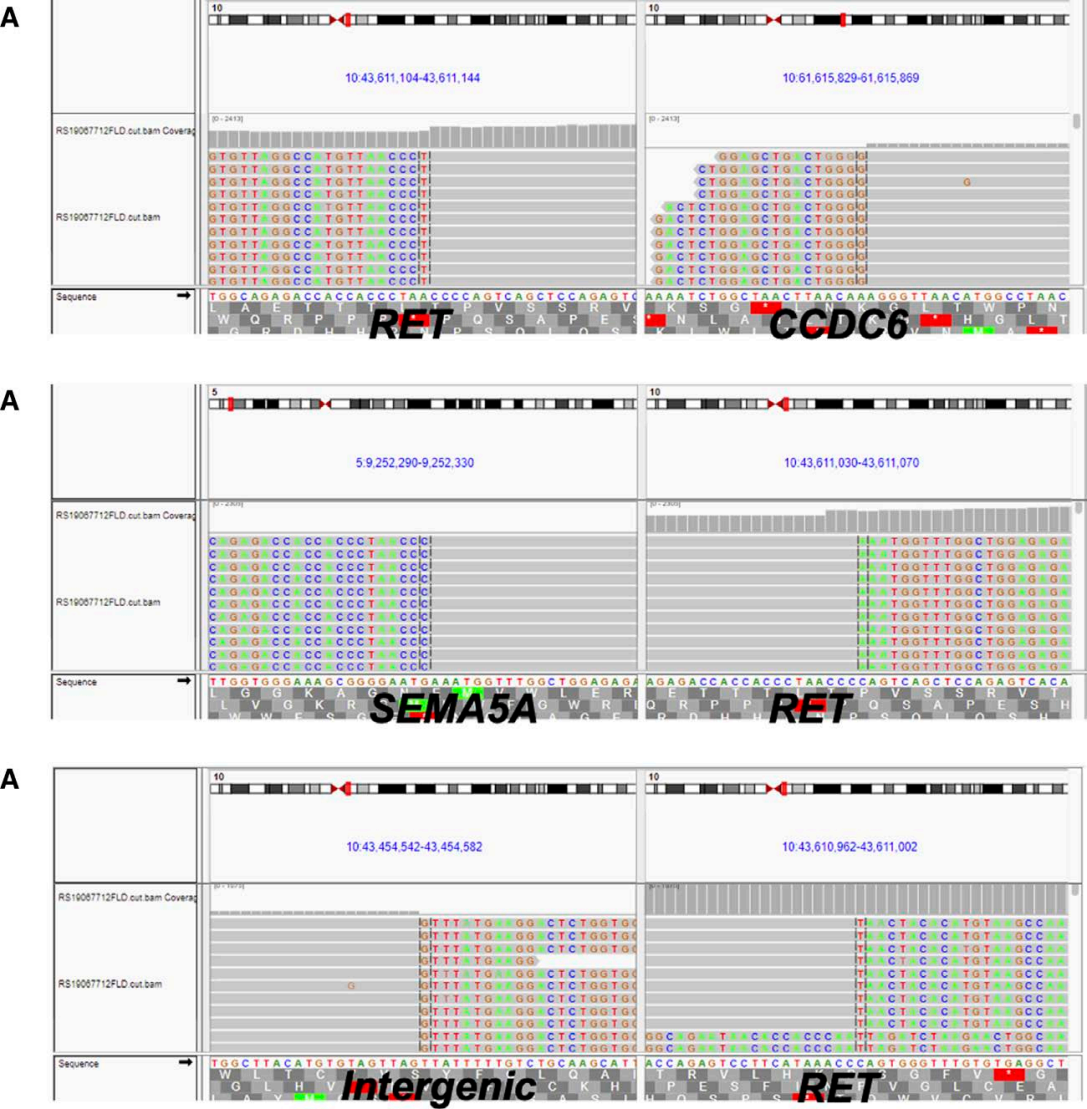
The IGV picture representing the CCDC6-RET (A), SEMA5A-RET (B) and LINCO1264-RET (C) fusions in the pleural effusion sample.

**Figure 3.**
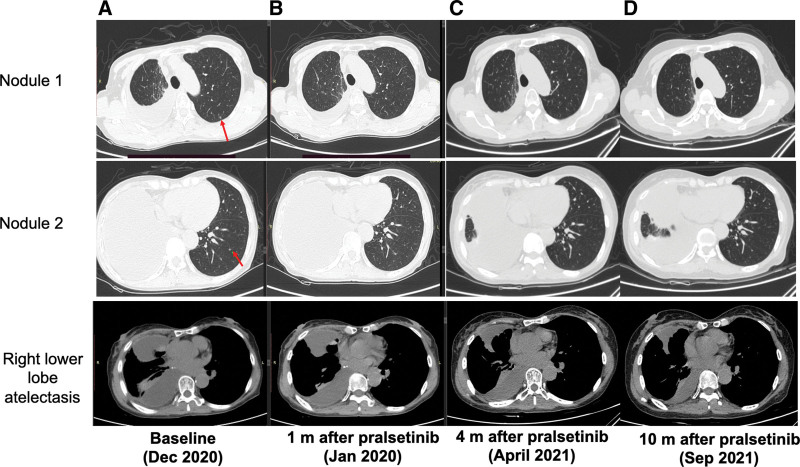
Tumor response to pralsetinib. A baseline before treatment. Red arrows refer to micronodulars. (B) 1 mo after pralsetinib treatment, multiple nodules disappeared, the region of right lower lobe atelectasis shrank, pleural fluid reduced. (C) 4 mo after pralsetinib treatment, patient remained as partial response. (D) 10 mo after pralsetinib treatment patient remained stable.

In December 2019, next-generation sequencing was performed on pleural effusion sample of patient using a 520-gene panel (Burning Rock Biotech, Guangzhou, China) and identified 3 concurrent *RET* gene fusions: *CCDC6-RET* (C1:R12) (allele frequency [AF]: 38.67%), *LINCO1264-RET* (intergenic: R12) (AF: 24.95%) and *SEMA5A-RET* (S5:R12) (AF: 6.05%) (Fig. 2A–C). In June 2020, the patient initiated the 4th line treatment with lenvatinib (8 mg qd), CT scan showed stable disease for 6 months then disease progressed suggested by the increased volume of pleural effusion and the emergence of multiple pulmonary micronodules (Fig. 3A).

In December 2020, the patient signed informed consent form, pralsetinib (GAVRETO, Blueprint Medicines Corporation) was administrated with an initial dosage of 400 mg qd. Due to creatinine increased to 220 umol/L and a deficient creatinine clearance rate (20 mL/min), the pralsetinib dosage was gradually reduced as recommended (400 mg-300 mg-200 mg qd) and pralsetinib 200 mg was administrated intermittently from March 2021 to July 2021, since July 2021 The patient continued to take 100 mg pralsetinib orally the creatinine remained at 180 umol/L.

At the time of the first follow-up in Jan 2021 the patient had achieved a PR, CT scan showed disappearance of micronodules, reduction of right pleural effusion and re-expansion of the right lower lobe (Fig. 3B). The pralsetinib reduced to 200 mg the patient remained as PR as follow-up in April 2021 (Fig. 3C). Second next-generation sequencing was performed with her pleural effusion sample and revealed the retaining of the 3 RET fusions but with declined abundance: CCDC6-RET (AF: 2.30%), LINCO1264-RET (AF: 2.53%) and SEMA5A-RET (AF: 0.60%) (Fig. 1). Although the patient took pralsetinib 100 mg orally since July 2021, Chest CT scan was showed the disease was stable in Sep. 2021(Fig. 3D). No distant metastasis of other organs was found, progressive disease free was 10 months.

The patient’s creatinine was 182 umol/L on baseline, then increased to 220 umol/L after pralsetinib oral administration, required dose reductions from 400 to 300 to 200 to 100 mg. Adverse events decreased after dose reduction to 100 mg of pralsetinib, and were primarily grade 2 according to the CTCAE5.0. In addition, the patient had grade I proteinuria, hypertension and rash. Hypertension and rash are relieved after pralsetinib reduction, and proteinuria persists, which may be related to basic disease of renal insufficiency. In addition, patient didn’t have increased count of eosinophils.

## 3. Discussion

In NSCLC, more than ten *RET* fusion partners have been described,^[3,6]^ among which *KIF5B-RET* occurs the most frequently with the prevalence ranging from 40% to 72%,^[1,7]^ followed by *CCDC6* (10-25%). Other identified partner genes include *TRIM33, ZNF477P, ERCC1, HTR4, CLIP1* etc.^[8]^ In the ARROW study, 13 cases had other or unknown types of RET fusion, accounting for 10.7%, but no specific fusion partner and its efficacy were reported.^[9]^ In this case, we identified 2 novel *RET* fusions *LINCO1264-RET* and *SEMA5A-RET* co-occurring with the common *CCDC6-RET*. *LINCO1264-RET (intergenic: R12*) results in the exon 12 of *RET* gene 3’-juxtaposed with an intergenic region, while *SEMA5A-RET* (S5:R12) is predicted to produce an in-frame fusion of *SEMA4* exon 5 with *RET* exon 12. Both novel fusions retain the intact RET kinase domain thus might be potential oncogenic drivers.

After the detection of *RET* fusions, the patient first received the treatment with a multikinase inhibitor lenvatinib, but the efficacy was limited, it similar with outcome of clinic trials, In phase 2 trials, cabozantinib and lenvatinib showed low response rates (ORR: 16%–28%, mPFS: 7.3 months).^[9]^ A retrospective multicenter registry analysis showed response rates ranging from 18% to 37%.^[10]^ Owing to the low activity and toxicity concerns with multikinase inhibitors, it is not recommended for the treatment of NSCLC with *RET* fusion.

Luckily with the recent approval of the new generation of selective RET TKIs, the patient switched to pralsetinib and showed an immediate response. Moreover, allele frequencies of all 3 fusions declined in the pleural effusion as the PR achieved, suggesting the responsiveness of all 3 of them to pralsetinib including the 2 novel ones. In our database, only 1 patient with 3 RET fusions was found, and the abundance decreased significantly after treatment. In the phase ARROW trial, pralsetinib demonstrated a promising ORR of 60% and DCR of 93% in *RET*-rearranged NSCLCs.^[10]^ Most patients had a duration of response ≥ of 6 months.^[9]^ So far, we have not found report about *LINCO1264-RET* and *SEMA5A-RET* co-occurring with the common *RET* was sensitive to pralsetinib or LOXO 292 (selpercatinib) in the NSCLC. Our case also showed that pleural effusion supernatant is an alternative liquid biopsy specimen for detecting rare *RET* fusion genes.^[11,12]^

Of note, this case had a history of chronic renal insufficiency. The pralsetinib was administrated with gradually reduced dosages (down to 200 mg qd) and eventually discontinued due to the persistent increase in creatinine. Reducing the pralsetinib dosage to 100 mg qd administrated creatinine was maintained at grade 2. Pralsetinib is primarily metabolized by liver enzyme CYP3A4 and to a lesser extent by liver enzyme CYP2D6 and CYP1A2, in vitro. It was predominately excreted in feces (approximately 73%). In addition, mild and moderate renal impairment (CLcr 30–89 mL/min) are safe on the exposure of pralsetinib. It has revealed a tolerable toxicity with most treatment-related adverse events (TRAEs) being grade 1 to 2, consisting of increased aspartate aminotransferase (31%), anemia (22%), increased alanine aminotransferase (21%), constipation (21%) and hypertension (20%), elevated blood creatinane (13%), no at grate 3 to 4. In the ARROW trial, 15% and 60% of NSCLC patients required permanent discontinuation of pralsetinib and dosage interruptions, respectively, due to adverse reactions. Dosage reduction was required in 36% of patients.^[9]^ In the last follow-up of our case, reducing the pralsetinib dosage to 100 mg qd the creatinine no further deterioration, and still have clinical benefits.

For the first time, we described a renal insufficient patient with NSCLC harboring 3 concomitant *RET* fusions, including 2 novel partners *LINCO1264* and *SEMA5A*. The patient failed to respond to lenvatinib but achieved a PR to the reduced dosage of pralsetinib accompanied with declined frequencies of all 3 *RET* fusions. Our case also indicates the responsiveness of the newly identified *RET* fusions to pralsetinib and highlights the necessity of dosage interruption especially in treating patients with background diseases.

## Acknowledgments

The authors thank Dr Lin Shao and Li Yan from Burning Rock Biotech for their assistance in manuscript writing and data analyzing.

## Author contributions

**Conceptualization:** Wenxiang Ji, Yuchen Han, Hong Jian.

**Data curation:** Linping Gu, Wenxiang Ji, Yunhua Xu.

**Investigation:** Wenxiang Ji.

**Methodology:** Yunhua Xu.

**Project administration:** Linping Gu.

**Supervision:** Yuchen Han, Hong Jian.

**Writing – original draft:** Linping Gu, Hong Jian.
